# The psychometric properties of the exercise dependence scale among South African university students

**DOI:** 10.17159/2078-516X/2025/v37i1a20698

**Published:** 2025-10-15

**Authors:** A Rathilal, RL Van Niekerk

**Affiliations:** Department of Psychology, University of Fort Hare, South Africa

**Keywords:** exercise addiction, maladaptive, questionnaire, factorial validity, reliability

## Abstract

**Background:**

Exercise dependence is a maladaptive pattern of exercise that can lead to physical and psychological impairment. This phenomenon can be assessed using the Exercise Dependence Scale (EDS-R). However, the reliability and validity of the scale have yet to be established for the South African context.

**Objectives:**

The purpose of this study was to verify the factorial structure and reliability of the Exercise Dependence Scale among South African university students.

**Methods:**

A cross-sectional study was conducted among 486 university students (176 males and 303 females, 4 non-binary and 3 participants not willing to disclose their gender) with a range of ages between 18 and 62 (23±6 years) who were invited to complete an online questionnaire comprising a demographic questionnaire and the EDS-R.

**Results:**

Exploratory factor analysis confirmed the seven-factor model of the EDS-R, with all subscales demonstrating acceptable to high internal consistency (α=0.75–0.94). Confirmatory factor analysis indicated good model fit indices (Comparative fit index = 0.96, Tucker-Lewis index = 0.95, Root mean square error of approximation = 0.06, 90% CI: 0.05–0.070, Standardised root mean square error residual = 0.05).

**Conclusion:**

Overall, the results of this study indicate an acceptable fit index, confirming the 7-factor structure of the EDS-R. However, it would be beneficial to remove item 10 from this scale for the South African content or consider rephrasing items 10 and 19. Further research is needed to investigate and verify the factorial structure of the EDS-R in the South African context.

It is widely agreed that individuals can become dependent and addicted to substances such as alcohol and drugs. However, individuals can also become addicted to certain objects and activities, such as shopping, eating, gambling, and exercising. The positive outcomes of exercise are irrefutable. Nevertheless, in some cases, exercise can have negative connotations and consequences when it becomes excessive. This excessive amount of exercise can lead to exercise dependence, a key area of concern in sports and exercise psychology. Exercise dependence can be depicted as a maladaptive sequence of exercise where moderate to intense physical activity becomes a compulsive behaviour.^[1,2]^

Research on exercise dependence is continuously emerging, with several studies focusing on developing theoretical models to explain the development of exercise dependence.^[2]^ A popular model explaining exercise dependence was developed by Hausenblas and Symons Downs^[1]^, who adapted the diagnostic criteria of substance abuse from the DSM-IV to develop this model, as shown in Table 1.^[3]^ A systematic review of exercise dependence within the last decade revealed 681 articles that investigated key facets of exercise dependence.^[4]^ Despite extensive research on exercise dependence, there has been only one attempt to develop a diagnostic criterion for this phenomenon. Currently, the DSM-V only includes gambling disorder as a non-substance-related disorder. However, several other conditions were considered for this category.^[5]^ Nevertheless, exercise dependence was not included in the DSM-V as a mental health disorder due to the lack of ‘peer-reviewed evidence to establish the diagnostic criteria’.^[6]^ Studies have shown that individuals at risk for exercise dependence show average levels of psychological morbidity and psychological distress. As such, the report findings are insufficient to warrant classifying exercise dependence as a disorder.^[7,8]^

There are two types of exercise dependence: primary and secondary. These two distinctions are used to separate excessive exercise as a distinct pathology from a related characteristic of an underlying eating disorder. Individuals with primary exercise dependency meet the criteria for exercise dependence and exercise solely for psychological gratification and fulfilment.^[2]^ On the other hand, secondary exercise dependence occurs when individuals with primary exercise dependence choose to exercise to achieve a separate outcome or goal, such as facilitating an eating disorder or body image dysfunction.^[9]^ With secondary exercise dependence, exercise is employed as a compensatory behaviour to compensate for weight loss, control body composition, and enhance physical appearance. Furthermore, secondary exercise dependence can lead to consequences such as the early onset of an eating disorder and lower body mass index.^[9]^

Several studies have investigated the prevalence of individuals at risk for exercise dependence. There are discrepancies in the prevalence of exercise dependence. For instance, studies have indicated that approximately 2–15% of regular exercisers are affected by exercise dependence.^[4,9]^ As this study will focus on a sample of South African university students, it is vital to highlight exercise dependence among university students. Studies have shown that the prevalence of university students at risk for exercise dependence is 3–10%.^[1,3,10]^

University students are particularly vulnerable to exercise dependence as they experience a significant transition from adolescence to adulthood, marked by profound physical and psychological changes.^[11,12]^ This phase underscores the importance of addressing students' physical and mental health challenges as they navigate increased independence and academic responsibilities.^[13]^ Within this context, psychological stress among university students is likely to intensify. Stress and its associated emotional responses, such as anxiety and depression, are closely linked to exercise dependence, with stress being identified as a key component in the etiological model of this condition.^[4]^

One of the challenges for researchers in this field involves the assessment of exercise dependence. Based on the criteria in Table 1, the Exercise Dependence Scale (EDS-R) was developed. It consisted of seven components: withdrawal effects (items 1, 8, 15), continuance (items 2, 9, 16), tolerance (items 3, 10, 17), lack of control (items 4, 11, 18), reduction in other activities (items 5, 12, 19), time (items 6, 13, 20) and intention effects (items 7, 14, 21).^[1,3]^ The EDS-R has been used in several studies that investigate exercise behaviours and dependency, and it is a valid, reliable, and appropriate multidimensional measure of exercise dependence.^[1,3]^

However, several questionnaires assess the risk of exercise dependence, which raises questions about whether these scales measure what they intend to do.^[4]^ While several studies have assessed the structural validity of the EDS-R using confirmatory factor analysis, many have found the subscale ‘Reduction in Other Activities’ to be problematic in terms of convergent validity and reliability.^[3,14,15]^ Furthermore, there have been studies that tested alternative models of the EDS-R. For example, Allerge and Therme^[16]^ found a six-factor model where the sub-scales ‘lack of control’ and ‘time’ formed a single factor, while Rowicka^[17]^ proposed a five-factor model where the subscales ‘time’ and ‘reduction in other activities’ merged, as well as ‘tolerance’ and ‘intention effects’. It is important to consider that Downs et al^[3]^ suggested a further examination of the items of ‘reduction in other activities’ as a necessity to ascertain if a modification is required and to examine the ‘conceptual relevance of the items on this scale’.

Other studies that assessed the psychometric properties of the EDS-R analysed construct reliability using average variance constructed (AVE), weighted omega scores or Cronbach’s alpha coefficient. Similar to studies that assessed structural validity, the subscale ‘reduction in other activities’ was problematic. AVE scores of less than 0.50 for the subscale ‘reduction in other activities’ were found in many studies, including Costa et al.^[15]^ (AVE = 0.44) and Downs et al. (2004) ^[3]^ (AVE = 0.56). Additionally, studies that utilised Cronbach alpha scores found weak internal consistency, i.e. scores inferior to 0.70, for ‘reduction in other activities’.^[3.15,16]^

While the EDS-R is a valid and appropriate measure of exercise dependence, it’s crucial to consider key cultural factors specific to the South African context that could potentially impact the scale’s reliability. In South Africa, cultural factors such as language diversity, cultural interpretations of items, and social desirability bias significantly impact the reliability of psychometric scales. With 11 official languages and widespread multilingualism, scales that have not been adapted for the South African context might lead to misinterpretations of certain concepts and reduced reliability. Furthermore, cultural variations in how concepts such as behavioural norms are understood can result in inconsistent responses, as items may not carry the same meaning in different contexts.^[18]^ Social desirability bias further complicates reliability, as individuals may provide socially acceptable answers instead of truthful ones, especially on sensitive topics such as gender roles or mental health.^[19]^ This study used the EDS-R to assess ED among South African university students. Based on a comprehensive search using search engines such as Google Scholar, Ebsco Host, and Scopus, it has been concluded that the EDS-R has not been validated in the South African context. However, it has been used in postgraduate research. ^[20]^ As such, this study aims to assess the factorial validity and reliability of the EDS-R among South African university students.

## Methods

### Research design and population

A quantitative, cross-sectional and descriptive research design was conducted to assess the psychometric properties of the EDS-R among South African university students. A research sample size of at least 385 students from ten different universities was expected to meet the study's objectives at a 95% confidence interval, with an alpha level set at 0.05. In this study, a convenience sample of 486 students from ten South African universities completed the online questionnaire.

### Data collection

Research participants were directed to an online research platform (SurveyMonkey), where they were asked to complete a set of biographical questions and the EDS-R online once they provided informed consent. The socio-demographic questions included a range of questions about age, gender, educational background, intensity and frequency of exercise, and the amount of exercise. Participants were recruited in two ways: first, through an advertisement for the research study placed on university noticeboards and second, by approaching coaches from university sports teams to distribute an advertisement for the study among team members. The advertisement included relevant information about the study and a link to the online questionnaire.

Exercise dependence was assessed using the revised exercise dependence scale (EDS-R).^[1]^ The EDS-R is a 21-item scale that is based on the criteria for substance abuse from the DSM-IV and is scored similarly. Each item is scored on a 6-point Likert scale ranging from 1 (never) to 6 (always). A higher total exercise dependence score indicates a greater severity of exercise dependence symptoms. The EDS-R consisted of seven sub-scales: tolerance, withdrawal effects, lack of control, intention effects, time, reduction in other activities and continuance. The scale was used to categorise participants into one of three groups: a group at-risk for exercise dependence, a non-dependent symptomatic group, and a non-dependent asymptomatic group. Participants were classified based on their responses to the seven DSM-based criteria, with individuals scoring 5 or 6 on three or more criteria categorised as at risk for exercise dependence. Those scoring in the range of 3 to 4 on at least three criteria were classified as non-dependent symptomatic (i.e., display symptoms of exercise dependence but are not at risk), while participants scoring in the 1 to 2 range on at least four criteria were categorised as asymptomatic. Downs et al.^[3]^ reported high reliability of the sub-scales of the EDS-R for tolerance (α=0.78), withdrawal (α=0.90), continuance (α=0.90), lack of control (α=0.82), reduction in other activities (α=0.75), time (α=0.86) and intention effects (α=0.89).

### Ethical considerations

The study procedures were approved by the Health Research Ethics Committee of the University of Fort Hare (REC-100118-054) and Northwest University (NWU-00084-23-S1). Research participants who met the study's criteria were invited to participate. All participants were informed about the aims and objectives of the study, their right to withdraw at any stage, and the rules regarding anonymity and confidentiality, which were necessary due to the online nature of the survey and the intended use of the data for research purposes.

### Statistical analysis

Data analysis was conducted using IBM SPSS Statistics (Version 29)^[21]^ to analyse descriptive statistics, exploratory factor. analysis and internal consistency and the Lavaan package in R (version 4.4.1) for confirmatory factor analysis (CFA).

Preliminary data analysis and initial data screening were conducted to verify statistical assumptions of normality and to code data. The EDS-R scores were summed to determine the scale and sub-scale scores. Descriptive and inferential statistics were calculated to determine the sample's frequencies, mean, and standard deviation scores.

To assess the factor structure, exploratory factor analysis using principal component analysis (PCA) was used. To verify the reliability of the EDS-R, the inter-item correlation matrix and Cronbach’s Alpha scores were used as measures of internal consistency. CFA was used to assess the measurement structure of the constructs and determine the appropriateness of the items of the EDS-R. The maximum likelihood estimation method was used to estimate the fit parameter of the CFA model.

The chi-square goodness-of-fit statistic (χ^2^), the root mean square error of approximation (RMSEA), the standardised root mean square error residual (SRMR), the comparative fit index (CFI), and the Tucker-Lewis index (TLI) were used to assess the model's fit indices. CFI and TLI > 0.95, RMSEA < 0.06, and SRMR < 0.08 indicate a good model fit.^[22]^

## Results

### Descriptive statistics

The study sample included 486 participants: 176 males, 303 females, 4 identifying as non-binary, and 3 participants who chose not to disclose their gender. Their ages ranged between 18 and 62 (23±6 years; mean±standard deviation). Results confirmed that 28% of the sample met the World Health Organisation’s guidelines for physical activity and exercise, with an average training session of 61±45 minutes and 1.9±0.7 minutes of moderate to vigorous exercise intensity per day. Furthermore, 10% of the participants were at-risk of exercise dependence, 59% were non-dependent symptomatic, and 30% were non-dependent asymptomatic. More information on the mean scores and standard deviations of the sub-scales of the EDS-R is presented in Table 2.

### Exploratory factor analysis and reliability of the Exercise Dependence Scale

The EDS-R^[1]^ was subjected to PCA, which was used as the extraction method. Before performing PCA, the factorability of this scale was assessed by reviewing the data. The correlation matrix revealed several correlation components with correlation coefficients of 0.3 or higher. The Kaiser-Meyer-Olkin value was 0.92, which exceeded the ideal value of 0.6^[24]^, while Bartlett’s test of Sphericity was significant (p<0.001), indicating suitable data for factor analysis.

In this study, PCA was done in two steps. In step one, PCA indicated the presence of five components with eigen values exceeding one. In step two, PCA was conducted whilst extracting a fixed number of seven factors to align with the EDS-R scale. Using Catell’s scree test^[24]^, it was decided to retain seven components for further investigation. Oblim with Kaiser normalisation was the rotation technique that was performed to assist in the interpretation of these seven components. A summary of this step is provided in Table 2 below. Cronbach alpha scores for all seven factors of the EDSR scale exceeded 0.70, indicating good internal consistency. However, including item 12 in the ‘reduction in other activities’ factor decreased the score to α=0.75.

### Confirmatory factor analysis

The CFA results of a chi-square statistic for the user model indicate a poor fit (χ^2^(148)=345, df=148, p<0.001). However, the chi-square statistic can be sensitive to sample size, prompting further evaluation through comparative fit indices and other fit measures. The results of the model fit indices (CFI=0.96, TLI=0.95, RMSEA=0.06, 90% CI: 0.05–0.07, SRMR=0.05) suggest that the model provides an acceptable representation of the data despite the significant chi-square value. More information on the output of CFA can be found in Table 3.

The standardised factor loadings of all factors in the EDS-R were calculated. All items demonstrated significant loadings on their respective factors, except for item 10 (λ=−0.05, p=0.54), which suggests that this item is not well-represented in the symptom of tolerance in the EDS-R. This suggests that item 10 may require revision or removal from the EDS-R to improve validity and reliability.

The ‘reduction in other activities’ subscale demonstrates acceptable factor loadings for all its items. However, item 19 had the lowest factor loading, suggesting some conceptual overlap with other dimensions of exercise dependence or potential inconsistencies in the interpretation of this subscale. This suggests the need to evaluate whether this subscale accurately represents the intended construct and whether adjustments or improvements to the items are required.

## Discussion

This study aimed to examine the factorial validity and reliability of the EDS-R administered to a sample of South African university students. The initial factor analysis showed a five-factor model of the EDS-R, where the subscales ‘intention effects’ merged with ‘tolerance’ and ‘lack of control’ merged with two items of ‘time’. In the initial analysis, items 12 (from ‘reduction in other activities’) and 6 (from ‘time’) were removed from the model. Similar issues with the subscales ‘intention effects’, ‘tolerance’, ‘reduction in other activities’ and ‘time’ were noted in other studies.^[16,17]^ This study accepted a seven-factor model solution that aligned with the EDS-R. However, a few issues arose regarding the ‘reduction in other activities’.

Firstly, item 12 loaded on ‘time’. Secondly, this subscale had the lowest score for internal consistency, which corroborates other research findings.^[15,16,17]^ The consistent issues with ‘reduction in other activities’ have led to the scrutiny of the wording of the items. Specifically, Deflandre and Kassabian^[14]^ question whether exercise dependence is a choice or a preference concerning items 5 and 19. To weaken the idea of time from this subscale, these authors suggest reforming items 5 and 19 to “I exercise so much that I cannot spend time on family activities/friendly activities/occupational activities or studies”.^[14]^

The results also indicated a negative factor loading for the subscales ‘reduction in other activities’, ‘lack of control’ and ‘time’. Generally, these results would signify an inverse relationship between the factor and the observed variable. Scores with a negative factor loading would be reverse-scored for psychometric scale development. However, reverse-scoring items in the EDS-R would prove problematic, as all items are phrased to reflect exercise dependence symptoms directly. As a result, reverse scoring would distort the intended meaning, creating inconsistencies with the diagnostic criteria of exercise dependence. For example, reverse-scoring item 6 (‘I spend a lot of time exercising’) would paradoxically contribute less to the total exercise dependency score, contradicting the construct of exercise dependence.

As a result, a more comprehensive approach is needed beyond reverse scoring. Therefore, a thorough examination of the problematic items and factors is essential. This necessitates qualitative exploration, such as cognitive interviews or focus groups with the target South African population, to understand interpretations of items in the EDS-R. Concurrently, employing Rasch analysis (or other Item Response Theory models) can provide objective psychometric evidence. This can statistically identify item misfit, detect Differential Item Functioning (DIF) across language groups, evaluate the logic of the response scale, and assess the unidimensionality of each subscale construct.

Based on research findings, two items of the EDS-R warrant attention. Item 10 showed a negative factor loading on the Tolerance subscale, which may suggest that it poorly captures the construct’s essence, i.e., escalating effort to achieve desired effects. This may reflect cultural differences in how exercise frequency is perceived. For instance, South African populations might prioritise intensity or duration over frequency due to socioeconomic constraints (e.g., limited access to facilities or only exercising two to three times per week due to other obligations). As such, it would be beneficial to remove item 10 from the scale or rephrase the item using alternative terminology such as ‘I continually exercise longer to achieve the desired effects/benefits’. Although item 19 was significant in the CFA, it showed a low factor loading on the Reduction in Other Activities subscale. This can be due to ambiguous phrasing, as ‘I choose to exercise so that I can get out of spending time with family/friends’ might not fit South African society, which values collectivism, familial obligations and group cohesion. As such, deliberate cultural and linguistic adaptation of the scale would be beneficial. This can involve expert translation and rigorous back-translation into major local languages with input from bilingual experts and target group representatives. Based on qualitative and Rasch findings, problematic items would need specific rewording to retain core symptom meaning while enhancing cultural appropriateness. For instance, adopting Deflandre and Kassabian's^[14]^ suggestion for Items 5 and 19 would remove the problematic "choice" implication.

Additionally, it is essential to note that the English version of the EDS-R was administered to the sample in this study. South Africa is a multilingual country, where the most prominent first or home languages are isiZulu (25%), isiXhosa (15%), and Afrikaans (12%).^[24]^ The negative factor loadings of the three subscales suggest that these items may be interpreted differently. For example, in the isiZulu language, several phonetic sounds aren’t used in English. The pronunciation of certain words and phrases can have cultural implications that may lead to barriers to understanding and interpretation.^[25]^ For individuals whose first language isn’t English, this could potentially mean that items from subscales with negative factor loadings were misinterpreted. For example, item 5, when translated into Zulu, can be read as ‘ngingathanda ukuzivocavoca kunokuba ngichithe isikhathi nomndeni’. The words ‘ngichithe isikhathi’ mean ‘I spent time’. However, colloquially, it can also be interpreted as ‘I wasted time’. As a result, item 5 would mean ‘I would rather exercise than waste time with family/friends’. Therefore, individuals might interpret the item as criticising family time rather than measuring prioritisation of exercise. This could skew the results, especially in cultures where family bonds are highly valued, making the item culturally loaded rather than neutral. Further research, including Rash Modelling, is suggested to address the issue.

The seven symptoms of exercise dependence in the EDS-R are based on the diagnostic criteria of substance abuse from the DSM-IV.^[1]^ However, it is vital to consider that the criteria for substance abuse in the DSM-V groups the symptoms of time, intention effects and lack of control into one group termed as impaired control and reduction in other activities within social impairment.^[6,14]^ Furthermore, the DSM-V has not included exercise dependence as a mental health disorder. However, it has been suggested that the changes in the criteria of substance abuse in the DSM-V and the inclusion of behavioural components allow room for clarification in conceptualising exercise dependence.^[14]^ Overall, it can be concluded that the seven sub-scales of the EDS-R measure the construct of exercise dependence as defined in the DSM-IV criteria for substance abuse.

### Limitations and recommendations for future research

Although the research findings provide support for the psychometric properties of the EDS-R in a South African context, several limitations exist. Firstly, the cross-sectional research design restricts the ability to make causal inferences regarding exercise dependence among South African university students. As data were collected at a single point in time, it is challenging to determine how exercise dependence may develop or fluctuate over different stages of students’ lives. Secondly, the reliance on self-reported data introduces potential biases, including social desirability and inaccurate recall, as participants may overestimate or underestimate their exercise behaviours. In the context of exercise dependence, individuals might underreport problematic exercise behaviours to avoid stigma or overreport them to conform to societal ideals of fitness and health. Thirdly, the sample used in this study consists solely of South African university students. This limits the generalizability of the findings, as the results of this study may not apply to broader populations, such as non-student groups, individuals from diverse cultural backgrounds, or various age cohorts. Additionally, the online distribution method may have inadvertently excluded students with limited internet access, potentially resulting in sampling bias.

In light of these limitations, several recommendations are proposed for future research. Firstly, future studies could employ a longitudinal research design to provide insights into the development and changes in exercise dependence over time, thereby allowing for stronger causal inferences. Additionally, administering the EDS-R at multiple time points will allow for the assessment of the scale’s stability and reliability over time. Secondly, utilising test-retest reliability will allow researchers to reevaluate participants’ responses to the problematic sub-scales. Thirdly, broadening the sample to include non-student populations and individuals participating in different modes of physical activity would enhance the generalizability of the findings. Alternatively, assessing the EDS-R with respect to criterion, discriminant, and convergent validity will assist in validating the scale for the South African context. Finally, employing a mixed-methods approach, including qualitative methods such as focus groups or interviews, would offer richer, in-depth insights into students’ experiences with exercise dependence and complement the quantitative data.

## Conclusion

The research findings of this study confirm the seven-factor structure of the EDS-R. Due to negative factor loadings on the subscales ‘Reduction in Other Activities’, ‘Time’ and ‘Lack of Control’, it would be beneficial to remove item 10 from this scale for the South African content or consider rephrasing items 10 and 19. Psychometric scale development is an ongoing process. As such, these findings contribute to the ongoing validation of the EDS-R as a reliable instrument for assessing exercise-related behaviours and their potential psychological implications.

## Figures and Tables

**Table 1:** Criteria for exercise dependence

Exercise dependence is a maladaptive pattern of excessive exercise behaviour that manifests in physiological, psychosocial, and cognitive symptoms. the following seven criteria for exercise dependence were adapted from the DSM-IV criteria for substance dependence.^[1,3]^	

1.	Tolerance is defined as either a need for increased amounts of exercise to achieve the desired effect or diminished effect with continued use of the same amount of exercise.
2.	Withdrawal effects is manifested by either the characteristic withdrawal symptoms fir exercise or the same amount of exercise is engaged in to relieve it avoid withdrawal symptoms.
3.	Intention effects represent when exercise is taken in larger amounts or over a longer period than was intended
4.	Lack of control is defined as a desire or unsuccessful effort to cut down exercise
5.	Time represents a greater deal of time spent in activities necessary to obtain exercise
6.	Reduction in other activities assesses social, occupational, or recreational activities are given up or reduced because of exercise.
7.	Continuance represents exercise that is continued despite knowledge of having a persistent or recurrent physical or psychological problem that is likely to have been caused or exacerbated by the exercise (e.g. continued running despite injury).

**Table 2:** Pattern structure matrix for principal component analysis (PCA) with Oblimin Rotation of Seven-Factor Solution of Exercise Dependence Scale (EDS-R)

Component Matrix of the EDS-R	IntE	Cont	WithE	Red	LoC	Tol	Time

7. I exercise longer than I intend	0.90						
14. I exercise longer than I expect	0.88						
21. I exercise longer than I plan	0.90						
2. I exercise despite recurring physical problems		0.82					
9. I exercise when injured		0.82					
16. I exercise despite persistent physical problems		0.89					
1. I exercise to avoid feeling irritable			0.87				
8. I exercise to avoid feeling anxious			0.87				
15. I exercise to avoid feeling tense			0.81				
5. I would rather exercise than spend time with family/friends				−0.79			
19. I choose to exercise so that I can get out of spending time with family/friends				−0.95			
4. I am unable to reduce how long I exercise.					−0.67		
11. I am unable to reduce how often I exercise					−0.53		
18. I am unable to reduce how intense I exercise.					−0.91		
3. I continually increase my exercise intensity to achieve the desired effects/benefits						0.78	
10. I continually increase my exercise frequency to achieve the desired effects/benefits						0.83	
17. I continually increase my exercise duration to achieve the desired effects/benefits.						0.76	
6. I spend a lot of time exercising.							−0.85
13. I spend most of my free time exercising							−0.77
20. A great deal of my time is spent exercising							−0.78
12. I think about exercise when I should be concentrating on school/work							−0.62

**Descriptive statistics of the sample**							

Eigen Value	9.5	1.7	1.4	1.3	1.1	0.93	0.8
Variance	45.3	8.1	6.7	6.4	5.2	4.5	3.9
Mean	7.7	7.5	10.7	4.2	6.9	10.9	9.7
Standard Deviation	4.6	4.3	4.6	2.6	3.7	4.7	5.2
Reliability	0.94	0.86	0.86	0.80	0.78	0.89	0.88
Rotates Sum of Squares	5.9	4.8	5.1	3.8	4.0	4.8	6.7

IntE, intention effects; Cont, continuance; WithE, withdrawal effects; Red, Reduction in other activities; LoC, Lack of control; Tol, tolerance

**Table 3:** Confirmatory factor analysis of the Exercise Dependence Scale (EDS-R)

Item	Est	SE	Z-value	p (>|z|)	Std. load	Std est	λ	p-value

**Withdrawal effects**								

Item 1	1.00				1.20	0.71	1.20	< 0.001*
Item 8	1.22	0.08	14.5	0.000	1.46	0.09	1.46	< 0.001*
Item 15	1.35	0.09	15.6	0.000	1.61	0.93	1.61	< 0.001*

**Continuance**								

Item 2	1.00				1.38	0.9	1.38	< 0.001*
Item 9	0.84	0.05	15.8	0.000	1.15	0.8	1.15	< 0.001*
Item 16	1.08	0.06	17.5	0.000	1.49	0.9	1.49	< 0.001*

**Tolerance**								

Item 3	1.00				1.36	0.78	1.36	< 0.001*
Item 10	−0.03	0.05	−0.6	0.543	−0.05	−0.03	−0.05	0.543
Item 17	1.12	0.07	15.2	0.000	1.53	0.87	1.53	< 0.001*

**Lack of control**								

Item 4	1.00				0.94	0.60	0.94	< 0.001*
Item 11	1.15	0.11	11.0	0.000	1.08	0.78	1.08	< 0.001*
Item 18	1.29	0.11	11.3	0.000	1.21	0.82	1.21	< 0.001*

**Reduction in other activities**								

Item 5	1.00				1.27	0.60	1.27	< 0.001*
Item 12	0.85	0.07	12.4	0.000	1.08	0.69	1.08	< 0.001*
Item 19	0.67	0.06	12.4	0.000	0.88	0.68	0.88	< 0.001*

**Time**								

Item 6	1.00				1.30	0.82	1.30	< 0.001*
Item 13	0.94	0.05	18.6	0.000	1.23	0.86	1.23	< 0.001*
Item 20	1.01	0.05	20.0	0.000	1.32	0.89	1.32	< 0.001*

**Intention effects**								

Item 7	1.00				1.51	0.93	1.51	< 0.001*
Item 14	1.03	0.05	19.7	0.000	1.56	0.94	1.56	< 0.001*
Item 21	0.94	0.05	18.8	0.000	1.43	0.88	1.43	< 0.001*

* indicates significance p<0.05;

Est, Estimate; SE, Standard Error; p(>|z|), p-value associated with z-value; Std. Load, Standardised loading; Std. est, standardised estimate; λ, Factor loadings
